# First report of the isolation and molecular characterisation of *Lactococcus garvieae* in dairy cattle in Poland

**DOI:** 10.2478/jvetres-2025-0035

**Published:** 2025-06-13

**Authors:** Henryk Krukowski, Andrzej Lisowski, Elżbieta Puacz, Witold Chabuz, Małgorzata Targońska-Karasek

**Affiliations:** 1 Department of Animal Hygiene and Environmental Hazards, 20-950 Lublin, Poland; 2 Department of Cattle Breeding and Genetic Resources Conservation, University of Life Sciences in Lublin, 20-950 Lublin, Poland; 3 Maria Sklodowska-Curie Warsaw Higher School of Medicine, 00-136 Warsaw, Poland

**Keywords:** bovine mastitis, pathogen, *Lactococcus garvieae*

## Abstract

**Introduction:**

*Lactococcus garvieae* is a catalase-negative, Gram-positive coccus that occurs in pairs and short chains. It is present throughout the world and a causative agent of bovine mastitis. However, prior to writing, no case of mastitis caused by this bacterium had been reported in Poland. The aim of this research was to investigate the presence of *L. garvieae* in milk samples from Polish cows with inflammation of the mammary gland.

**Material and Methods:**

A total of 118 milk samples from 96 Holstein-Friesian cows were spread on blood agar plates for microbial analyses. Then analysis with matrix-assisted laser desorption/ionisation–time-of-flight mass spectrometry, biochemical identification and antibiotic susceptibility evaluation in the VITEK 2 system and a PCR with primers compatible with 16S ribosomal RNA of *L. garvieae* were performed.

**Results:**

The presence of three strains of *L. garvieae* was confirmed in milk samples. Analysis of antibiotic susceptibility showed the susceptibility of all isolates to moxifloxacin, erythromycin, linezolid, teicoplanin, vancomycin, tetracycline, tigecycline, chloramphenicol and trimethoprim-sulfamethoxazole. *Lactococcus garvieae* had high resistance to benzylpenicillin, clindamycin and rifampicin. One of the isolated strains was additionally resistant to tetracycline and had different minimum inhibitory concentrations.

**Conclusion:**

To the best of our knowledge, *L. garvieae* had not been identified as an aetiological factor of mastitis in cows in Poland before, and the presented research is the first report.

## Introduction

*Lactococcus garvieae* is a catalase-negative, facultative anaerobic, Gram-positive coccus and is present throughout the world (15). It was identified and described for the first time in the early 1980s as a causative agent of bovine mastitis (3, 14). Previously, it was described as *Streptococcus garvieae* (3) and was later reclassified to the *Lactococcus* genus (23). Since then, numerous cases of *L. garvieae* infections in fish colonies as a highly opportunistic pathogen have been reported frequently in the literature (30).

This microorganism is a nonmotile, nonspore-forming bacterium that occurs in pairs and short chains. It can cause α-haemolysis on blood agar. The optimal growth temperature for this bacterium is 37°C, but it can grow between 10°C and 42°C, and some strains can grow weakly and slowly even at 45°C (17). These bacteria grow at pH 4.5–9.6 in broth containing 4% NaCl, although some strains can grow even at 6.5% NaCl (17). Because of these characteristics, it was misidentified as a new enterococcal species, *Enterococcus seriolicida*, when it was determined to be the pathogen responsible for septicaemia in yellowtail (*Seriola quinqueradiata*) (12, 17). However, subsequent phenotypic and genetic analyses comparing characteristics between the new species and isolates of *L. garvieae* showed that both organisms belonged to the same species, carrying the implication that *E. seriolicida* should be reclassified as *L. garvieae* (7, 8, 26).

The wide distribution of *L. garvieae* is most likely related to the ability of this bacterium to adapt and survive in many environmental conditions. It has been isolated from food and feed products, terrestrial waters and sewage (1), as well as from various aquatic and terrestrial animals (6, 11, 20, 27, 28). Like other widespread microorganisms, *L. garvieae* is phenotypically and genetically heterogeneous (8, 20, 29). Methods such as analysis of the acidification of sugars and the presence of the enzymes pyroglutamic acid arylamidase and N-acetyl-β-glucosaminidase have been used to identify and differentiate different biotypes of *L. garvieae* (29). Genotyping studies have also been performed, mainly on trout isolates. They have shown that *L. garvieae* forms clonal genetic groups (6, 20, 29). However, considerable genetic heterogeneity is observed among isolates from other hosts, environments or foods (20, 27). Recently, genetic and phylogenetic analyses have revealed genotypic differences supported by corresponding phenotypic variations, leading to the proposal of two subspecies: *Lactococcus garvieae* subsp. *garvieae* and *Lactococcus garvieae* subsp. *bovis* (12, 28)

*Lactobacillus garvieae* is known to be the causative agent of lactococcosis in various species of wild and farmed fish. Its proliferation peaks during summer, when the water temperature rises, and this also coincides with a rise in the number of *L. garvieae* infections in fish (30). The bacteria have also been isolated from cows and water buffaloes with subclinical mastitis (1, 3, 6, 13, 18, 21, 24, 26, 30) and pigs with pneumonia (27). In humans, *L. garvieae* is currently considered an opportunistic pathogen that causes a variety of infections (2, 22, 25). The increasing number of human infections in recent years has raised awareness of *L. garvieae* as an emerging pathogen associated with the handling or consumption of raw fish and seafood (2). However, the status of *L. garvieae* as a potential zoonotic bacterium is still contentious. Furthermore, the isolation of *L. garvieae* from faecal samples of healthy individuals (16) suggests that not all strains are pathogenic to humans or that not all humans are susceptible to *L. garvieae* infections.

Besides having been present in seafood, *L. garvieae* has also been found in some dairy products such as cheese and raw cow’s milk. The main objective of this research was to investigate and detect the presence of *L. garvieae* in milk samples obtained from cows with mastitis in Poland. To the best of our knowledge, this is the first report of mastitis in cows caused by *L. garvieae* in Poland.

## Material and Methods

The study was conducted on a dairy farm located in central Poland, keeping 366 Polish Holstein-Friesian dairy cows. The average milk yield of the cows was 11,673 kg of milk which contained 3.64% fat and 3.34% protein. The somatic cell count was 204,000 per mL. The animals were kept in a free-stall box barn and bedded with straw every day. The cows were milked in a 2 × 8 herringbone milking parlour over an average time per group of approximately 45 min. The cattle were fed in the total mixed ration system. The feed ration consisted of the following feeds: corn, grass and legume haylage, brewer’s and fermented corn grain, and soybean, rapeseed and cereal meal. A total of 118 quarter-milk samples which were positive in a California mastitis test (CMT) were collected from 50 cows with clinical (4 cows) and subclinical (46 cows) mastitis, following aseptic procedures as described by the National Mastitis Council (https://www.nmconline.org/nmc-protocols-guidelines-and-procedures). Milk samples were collected from quarters that scored 1, 2 or 3 in the CMT. Sample collection was performed aseptically, and samples were stored in sterile tubes under refrigerated conditions until microbiological and molecular analyses. For classical microbiological analyses, 10 mL of each milk sample was spread on blood agar plates with 5% defibrinated sheep blood. The plates were incubated at 37°C for 72 h, with observation every 24 h. After bacterial growth, colonies with single and pure growth were identified based on Gram stain, morphology and haemolysis patterns. Gram-positive, catalase-negative cocci were further evaluated using aesculin hydrolysis, haemolysis and the Christie–Atkins–Munch-Petersen reaction.

For three isolates suspected of being *L. garvieae*, additional identification analyses were performed. Milk from three cows suspected of being infected by *L. garvieae* was sampled again after 30 d and analysed with the procedures previously described. Additionally, analysis with matrix-assisted laser desorption/ionisation– time-of-flight (MALDI-TOF) mass spectrometry, biochemical identification in the VITEK 2 system and a PCR with primers compatible with 16S ribosomal RNA (rRNA) of *L. garvieae* were performed.

### Analysis of DNA

Before DNA extraction, bacterial isolates were transferred into Petri dishes with trypticase soy agar medium to multiply cultures, from which single bacterial colonies of 24-h cultures were taken for DNA isolation. A Bead-Beat Micro AX Gravity kit (A&A Biotechnology, Gdańsk, Poland) was used for DNA isolation according to the product protocol. The concentration and quality of the isolated DNA were evaluated using a NanoDrop One spectrophotometer (Thermo Scientific, Wilmington, DE, USA). The obtained samples of DNA were diluted with molecular-free water to 20 ng/μL for further analyses.

For molecular identification of bacteria, a primer pair was used amplifying a fragment of the 16S rRNA sequence. The sequence of the forward primer was AGA GTT TGA TCC TGG CTC AG and that of the reverse primer was CGG TTA CCT TGT TAC GAC TT. All PCRs were performed in 25 μL volumes containing 20 ng of genomic DNA, 2× PCR Mix Plus Red (A&A Biotechnology) and 0.2 μM of each primer. Amplification was carried out in a Biometra TOne thermal cycler (Analytik Jena, Jena, Germany). The PCR cycling conditions were as follows: an initial denaturation step of 5 min at 95°C; 32 cycles of 30 s at 95°C, 40 s at 62°C and 45 s at 72°C; and a final extension of 5 min at 72°C. The length and quality of the PCR products were verified by electrophoresis with 1% agarose gel. After positive verification, the DNA products were sequenced by an external service (Genomed, Warsaw, Poland). The generated sequences were used to perform basic local alignment search tool (BLAST) analysis to determine the nearest phylogenetic neighbours against the NCBI GenBank database of validly published prokaryotic names (http://www.ncbi.nlm.nih.gov/genbank/).

### Identification using VITEK 2

The biochemical identification of microorganisms was performed with VITEK 2 (BioMerieux, Marcy l’Étoile, France). A bacterial suspension was prepared from an isolated colony in a clear polystyrene test tube filled with 3 mL of sterile saline. The turbidity of the suspension was adjusted to a McFarland standard of 0.5 with the help of a VITEK 2 DensiCHEK instrument. The time between the preparation of inoculum and filling of the card was always less than 30 min. Identification with the VITEK 2 compact system was performed according to the manufacturer’s instructions using the Gram-positive card.

### Antibiotic susceptibility testing (AST) with VITEK 2

The 0.5 McFarland bacterial suspension was diluted to 1.5 × 10^7^ CFU/ml in 0.45% saline. The AST-ST03 card used for streptococci contained benzylpenicillin, moxifloxacin, clindamycin, erythromycin, linezolid, teicoplanin, vancomycin, tetracycline, tigecycline, chloramphenicol, rifampicin and trimethoprim-sulfamethoxazole. The card covers the spectrum of streptococci of groups A, B, C and G and streptococci of the mitis group. The interpretation of minimum inhibitory concentrations (MICs) was based on the recommendations of the European Committee on AST breakpoint tables for interpretation of MICs and zone diameters (https://www.eucast.org/clinical_breakpoints). In order to interpret the drug susceptibility of the tested *L. garvieae* strains, the cut-off points used were those for individual antibiotics determined in the therapy of infections with for *Streptococcus* group G and *Str. mitis*. These two groups of streptococci were selected due to their relationship with *Lactococcus* sp. The antibiotic–microorganism combinations selected for evaluating the performance of the VITEK 2 Compact system in this study are shown in Table 3. The card was automatically filled by a vacuum device, sealed and inserted into the VITEK 2 reader-incubator module (with incubation temperature set to 35.5°C), and subjected to a kinetic fluorescence measurement every 15 min. The results were interpreted by the database, and final results were obtained automatically. All cards used were automatically discarded into a waste container.

### Identification using MALDI-TOF mass spectrometry

To verify and confirm the bacterial identification, the MALDI Biotyper Microflex LT/SH mass spectrometry system (Bruker, Billerica, MA, USA) was used with the MBT IVD library v.12 (Bruker) containing 4.194 species. For comparing the spectra with the library entries, MBT IVD Compass 4.2.100 software (Bruker) was employed. The standard identification protocol described by the manufacturer of the spectrometer was followed and α-cyano-4-hydroxycinnamic acid was the matrix.

## Results

Forty-one bacterial and fungal isolates were identified by classical microbiological analyses. The quantitative distribution of the identified strains was as follows: *Lactococcus* sp. (*L. garvieae*) 7.32%, coagulase-negative staphylococci (*Staphylococcus* spp., *S. epidermidis, S. chromogenes* and *S. xylosus*) 43.9%, coagulase-positive staphylococci (*Staphylococcus* spp. and *S. aureus*) 12.2%, *Streptococcus* spp. (*Str*. spp., *Str. dysgalactiae* and *Str. uberis*) 24.39%, *Enterococcus* sp. (*Enterococcus faecalis*) 2.44%, *Corynebacterium* sp. (*Corynebacterium bovis*) 2.44%, *Micrococcus* spp. 4.88% and yeasts (*Candida* sp.) 2.44% (Table 1).

**Table 1.** Groups of microorganisms identified in microbiological analyses

Isolates	Number (n)	% of all milk samples	% of all strains
*Micrococcus* spp.	2	1.69	4.88
*Enterococcus* sp.	1	0.85	2.44
*Streptococcus* spp.	10	8.47	24.39
*Lactococcus* spp.	3	2.54	7.32
*Corynebacterium* sp.	1	0.85	2.44
Coagulase-negative staphylococci	18	15.25	43.90
Coagulase-positive staphylococci	5	4.24	12.20
*Candida* sp.	1	0.85	2.44
Contamination	1	0.85	–
No growth	76	64.41	–
Total	118	100	100

*Lactobacillus garvieae* was identified biochemically and by MALDI-TOF mass spectrometry. In the VITEK2 system, the probability for all three strains was 95%, which corresponds to very good identification.

In the MALDI Biotyper the scores were 2.16, 2.29 and 2.40, which are trustworthy against the benchmarks: score values higher than 1.99 are indicative of secure to highly probable species identification, those between 1.7 and 1.99 indicative of probable genus identification and those between 0.0 and 1.69 signify unreliable identification.

The results of BLAST showed that in the case of all three bacterial DNA samples, the highest similarity was observed to the 16S rRNA sequence of *L. garvieae*. The query sequence coverage ranged between 91 and 95%, and the percentages of identical base pairs ranged from 85.16% for the sequence obtained from bacterial strain number 64 to 98.74% for the sequence obtained from bacterial strain number 5 (Table 2). The results of molecular identification confirm that the analysed bacterial isolates belonged to *L. garvieae*.

**Table 2.** Results of basic local alignment search tool analysis

Bacterial strain No.	Query coverage (%)	Identity (%)	GenBank sequence length (base pairs)	Species	Accession No.
5	95	98.74	1,471	*Lactococcus garvieae*	LC377166.1
13	91	97.21	1,233	*Lactococcus garvieae*	PP422542.1
64	88	85.16	895	*Lactococcus garvieae*	MT000064.1

Analysis of antibiotic susceptibility with VITEK 2 revealed that all *L. garvieae* isolates were susceptible to moxifloxacin, erythromycin, linezolid, teicoplanin, vancomycin, tetracycline, tigecycline, chloramphenicol and trimethoprim-sulfamethoxazole. *Lactobacillus garvieae* had high resistance to benzylpenicillin, clindamycin and rifampicin. One of the isolated strains was additionally resistant to tetracycline and had different MIC values. The results of antibiotic susceptibility are presented in Table 3.

**Table 3.** Antimicrobial agents used in the study and antimicrobial susceptibilities of *Lactococcus garvieae*

Antimicrobial agent	MIC range (mg/L)					
	Strain No. 5		Strain No. 13		Strain No. 64	
	MIC	Interpretation	MIC	Interpretation	MIC	Interpretation
Benzylpenicillin	0.5	R	1	R	0.5	R
Clindamycin	≤ 1	R	≤1	R	≤1	R
Linezolid	≤2	S	≤2	S	≤2	S
Teicoplanin	≤0.12	S	≤0.12	S	≤0.12	S
Vancomycin	0.5	S	0.5	S	0.5	S
Tetracycline	0.5	S	≤16	R	0.5	S
Tigecycline	≤0.06	S	≤0.06	S	≤0.06	S
Moxifloxacin	0.5	S	0.5	S	0.5	S
Chloramphenicol	4	S	2	S	4	S
Rifampicin	≤4	R	≤4	R	≤4	R
Erythromycin	≤0.12	S	≤0.12	S	≤0.12	S
Trimethoprim-sulfamethoxazole	≤0.12	S	≤10	S	≤10	S

1 MIC – minimum inhibitory concentration; R – resistant; S – susceptible

## Discussion

Mastitis in cows is caused by the simultaneous occurrence and overlap of many unfavourable factors, of which the most important are pathogens. The aetiological agents that cause mastitis may vary depending on the climate, species or husbandry practices. These agents include a wide range of Gram-positive and Gramne-gative bacteria, mycoplasmas, fungi, yeasts, algae and viruses. *Lactococcus garvieae* is among these agents, but in addition is currently recognised as a species of clinical importance to human medicine besides being one to veterinary medicine (3–6).

In addition to being a factor responsible for mastitis in domestic and wild animals, *L. garvieae* is also an emerging pathogen in aquaculture as well as human endocarditis and sepsis. The pathogenic mechanisms of this bacterium are poorly understood (7), and the world literature on *L. garvieae* as an aetiological factor of inflammation of the mammary gland is also not extensive compared to the literature on other microorganisms. This may be due to the focus of most studies on major pathogens and the focus of rather limited counterpart studies on minor pathogens (8).

We confirmed the presence of *L. garvieae* in milk samples from three cows with symptoms of subclinical mastitis using three different methods. The general percentage of *L. garvieae* detected in milk samples was 2.54 (7.32% of all strains). It was comparable to the results of de Oliveira *et al*. (9), where 6.9% of 72 isolated bacteria were identified as *L. garvieae* using MALDI-TOF mass spectrometry.

The isolates identified in the presented study were susceptible to moxifloxacin, erythromycin, linezolid, teicoplanin, vancomycin, tetracycline, tigecycline, chloramphenicol and trimethoprim-sulfamethoxazole. However, they exhibited high resistance to benzylpenicillin, lincomycin and rifaximin, and one strain was resistant to tetracycline. A characteristic feature of *L. garvieae* is resistance to clindamycin, which distinguishes it from *Lactococcus lactis* (10). In our study, all three strains were resistant to clindamycin. These results are similar to those obtained by Xie *et al*. (11), with the difference that in our studies, all strains were resistant to penicillin. According to Scillieri Smith *et al*. (24), the odds of a successful bacteriological cure for bovine clinical mastitis were eight times higher when the agent was *Streptococcus dysgalactiae* or *Str. uberis* than when it was *L. lactis* or *L. garvieae*, indicating greater difficulties in treating diseases caused by *L. garvieae* than treating those caused by other bacteria.

To the best of our knowledge, *L. garvieae* had not been identified before the time of writing as an aetiological factor of mastitis in cows in Poland. It is possible that *Lactococcus* strains had already caused mastitis before our investigation, given the possibility that laboratories could have misclassified the cases as infections with *Streptococcus* spp. A similar hypothesis was presented by Rodrigues *et al*. (21).

## Conclusion

Our findings highlight the presence of *L. garvieae* as a bovine mastitis pathogen in Poland, a fact that had not been previously reported. The obtained results confirmed subclinical mastitis in cows caused by the identified pathogen. This underscores the importance of accurate identification and characterisation of pathogens to ensure effective treatment and management of mastitis.
